# Reuse of Drilling Waste Slurry as the Grouting Material for the Real-Time Capsule Grouting Technique

**DOI:** 10.3390/ma16041540

**Published:** 2023-02-12

**Authors:** Chenlei Jiao, Yu Diao, Gang Zheng, Yongchao Liu, Jianyou Huang, Ying Zhang, Lejun Zhao

**Affiliations:** 1 Key Laboratory of Coastal Civil Engineering Structure and Safety, Ministry of Education, Tianjin University, Tianjin 300350, China; 2 School of Civil Engineering, Tianjin University, Tianjin 300350, China; 3 Tianjin Municipal Engineering Design & Research Institute, Tianjin 300392, China

**Keywords:** real-time capsule grouting technique (RCG), drilling waste slurry grouting material, recycling, cement, bentonite, fly ash

## Abstract

A large amount of waste slurry is generated during construction, but direct sedimentation and transportation increase construction costs. Improper treatment leads to ecological and environmental pollution. This paper proposes to reuse drilling waste slurry (DWS) as a raw material from a particular project as a grouting material for the real-time capsule grouting technique (RCG) to replace cement grouting material. This not only deals with DWS but also solves the material demand of RCG. An orthogonal experimental design evaluated the performance of the DWS grouting material (DWS-GM). The five levels for the three factors of this experiment were selected, including the fluidity, bleeding rate, initial setting time, and compression strength. A linear model, support vector machines, and neural networks were used to construct regression models, and the effects of different contents of cement, bentonite, and fly ash on the DWS-GM performance were analyzed. The SVM regression model had better performance in describing the laws of fluidity, bleeding rate, and 28-day compressive strength. Furthermore, the optimization model is proposed to obtain the optimal formulation of the DWS-GM under specific constraints. The optimization results show that the optimal formulation of the DWS-GM was 5.6% cement and 6.9% bentonite. The BL, FL, IST, and 28DCS were 1.61%, 21.87 cm, 27.05 h, and 0.22 MPa to meet the functional requirements of the DWS-GM. The above research fully proves the feasibility of the DWS reuse application. We will further reuse DWS to develop other multifunctional material applications in combination with the control needs of RCG technology and technology from other fields.

## 1. Introduction

In recent years, with the increase in buildings and structures in the city, controlling the environmental impact of the deformation caused by construction is becoming increasingly critical. In response to the above problems, Zheng et al. proposed a theory of active deformation control that directly focuses on the protected object. By controlling the stress and deformation of the soil in critical areas, the integrated target control of the measurement and control of the protection object are realized [1,2]. A key technique in the active-control deformation method of the protected object is the real-time capsule grouting technique (RCG).

RCG can provide real-time active control, high efficiency, and precision in controlling the deformation of the protected object. This method has been well practiced in many projects [3,4,5,6]. RCG needs to use a lot of cement grouting material when activated. Previous research has optimized the traditional short setting time and the poor-fluidity materials used in this technique. A retarded cement grouting material with high fluidity and a low bleeding rate is prepared through the optimization of the material ratio and admixtures [7,8].

In addition, the slurry is considered a waste material. Its treatment has been an urgent problem to be solved during construction in recent years. It is not easy to spontaneously evaporate or precipitate due to the high-water content and viscosities. Currently, most of the waste slurry treatment methods are still traditional. The slurry pool is settled and then transported or landfilled. The problems faced by the conventional waste slurry treatment methods are as follows: (1) direct emissions lead to environmental pollution; (2) the slurry occupies a tense construction site; and (3) transportation and handling lead to high construction costs [9].

In the research of waste slurry treatment, the stability of the slurry conveying process has been improved by developing the corresponding coagulant material [10,11,12]. Further, many studies have proposed solidification technologies for waste slurry to enhance its stability and realize some reusability, such as geopolymers (blast-furnace cinder [13], calcium carbide residue, sodium silicate solution [14,15], lime, cement, and bitumen [16]) or additives for in situ solidification [17]. Waste slurry has been used in other fields, such as ceramic fabrication, according to the different chemical compositions [18,19,20]. Meanwhile, in construction engineering, grouting material was created by reusing the slurry (shield tunnels [21,22,23,24] and waste drilling [25]).

In other respects, many methods have also emerged to treat waste sludge, such as mechanical dehydration [26,27] and vacuum preloading [28,29,30]. In general, the treatment of waste slurry can be summarized as follows: (1) By optimizing the construction process, the utilization rate of materials can be improved, and the generation of waste slurry can be reduced. (2) The methods mentioned above summarizing some treatment measures are adopted to treat the waste slurry and then discharge it. (3) The characteristics of waste slurry are combined for treatment, recovery, and reuse.

Therefore, facing the massive demand of RCG for grouting materials, we developed new multifunctional materials based on engineering waste slurry that meet the application performance requirements of RCG. This way, the waste slurry in a part of the project is used. The trouble and costs caused by the slurry treatment are solved. Meanwhile, the grouting materials required for the startup of RCG are reduced to a certain extent, and only a few non-polluting common additive materials are needed to improve the waste slurry, which reduces the cost of RCG. In addition, RCG is set around the construction area or between the foundation pit and the transportation infrastructure from the current engineering application. The strength of the grouting material used in RCG should not be too high after consolidation, reducing the trouble when optimizing the urban infrastructure construction in the later stage. The analysis above shows that reusing DWS as a grouting material of RCG is a better option to reduce environmental effects and costs in construction.

## 2. Materials and Experimental Methods

### 2.1. Raw Material

**Figure 1:**
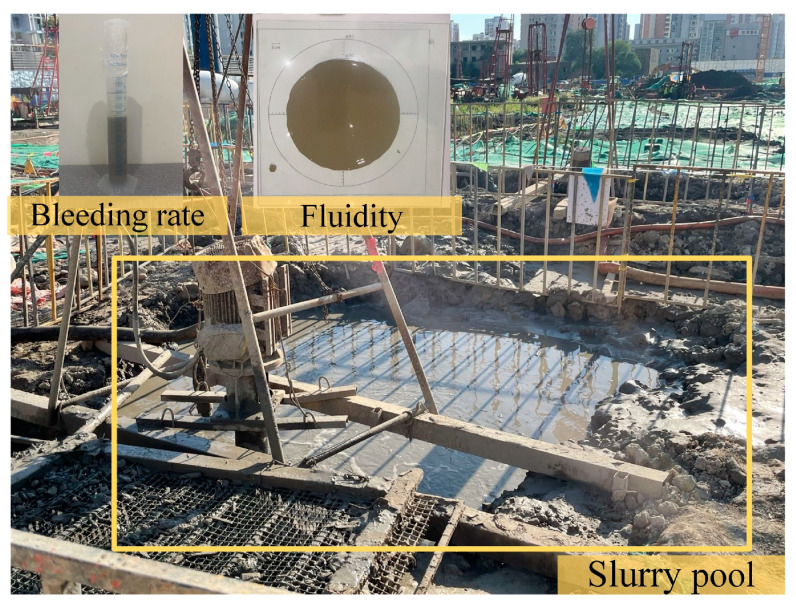
DWS from bored pile in the slurry pool.

(1) Drilling waste slurry (DWS): Drilling waste slurry (DWS), shown in Figure 1, was obtained from the bored pile construction in a particular project. The project site’s buried depths were mainly filled soil, muddy clay, silty soil, and silty clay within 25 m. The DWS would usually be put into a slurry pool for sedimentation and then transported. A large amount of DWS is created each year, and space is needed to store it. A common method is to separate water and soil particles, but dehydration usually costs a lot of energy. The main properties of DWS are shown in Table 1. The water content and bleeding rate of DWS are both very high.

**Figure 2:**
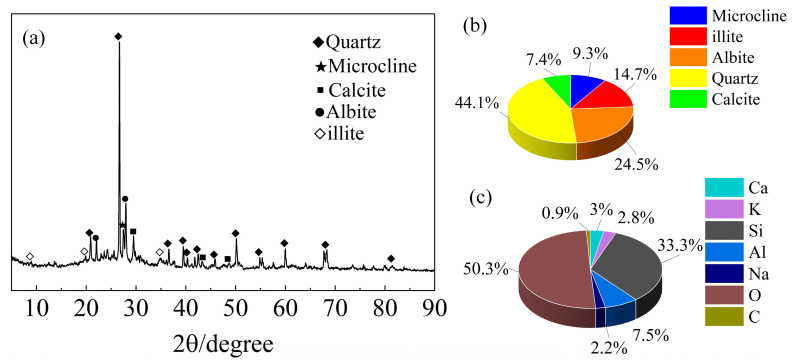
XRD and XRF analyses of DWS. (**a**) XRD spectra. (**b**) The mass fractions of ingredients. (**c**) The mass fractions of elements (Wt%).

The DWS was dried and ground into powder for scanning electron microscopy (SEM, step length: 0.02 degrees, rate: 4.25 degrees per minute), X-ray diffraction (XRD, voltage: 40 kV, current: 30 mA), and X-ray fluorescence (XRF) spectrometer analyses. The XRD analysis in Figure 2a showed that the DWS consisted mainly of microcline, illite, albite, quartz, and calcite. It also showed a wide range of features between 20 and 35°, demonstrating that the DWS predominantly consisted of crystalline structures. The weight percentages of the substances and elements are shown in Figure 2b,c.

**Figure 3:**
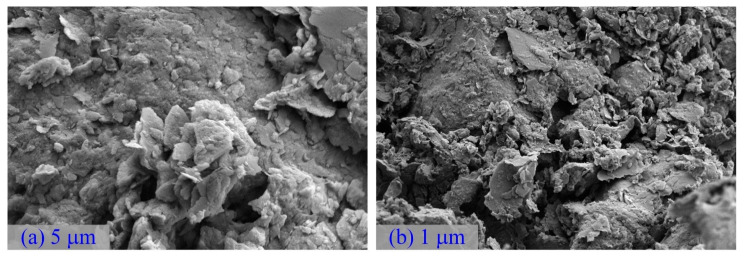
SEM images of DWS. (**a**) 2.0 KX (**b**) 10.0 KX.

**Figure 4:**
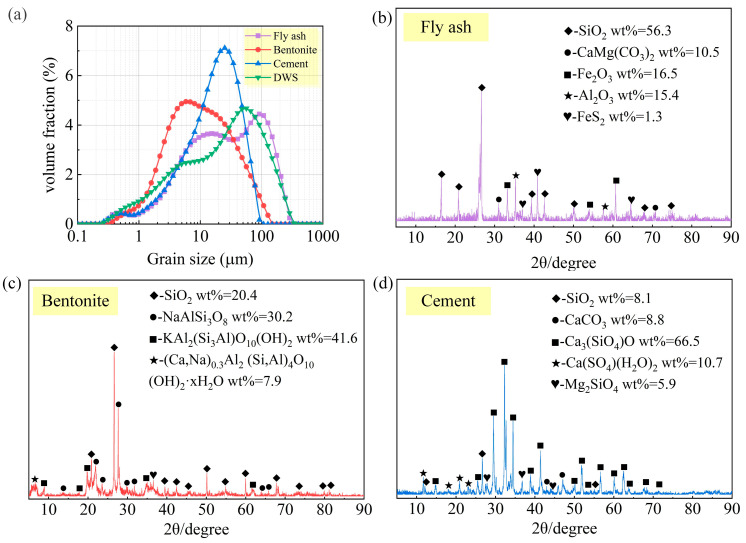
(**a**) Particle size distributions (**b**) XRD of FA (**c**) XRD of BE (**d**) XRD of CE.

SEM was conducted on the DWS specimens to study their morphological and microstructural features, as shown in Figure 3. They mainly comprised various two-dimensional flakes, massive particles with different shapes and sizes, and multilayer hierarchical structures. The two-dimensional flake structure provided a higher specific surface area. The particle size distribution of the DWS is shown in Figure 4a.

(2) Cement (CE): The cement utilized in this research was po42.5 Portland cement supplied by Weifang, Shandong, China. The main components of the CE are shown in Table 2. After curing for 3 days, the compressive and flexural strengths were 27.2 MPa and 5.5 MPa, respectively. After curing for 28 days, the compressive and flexural strengths were 42.5 MPa and 6.5 MPa, respectively. The final and initial setting times were 234 and 172 min, respectively.

(3) Bentonite (BE): The bentonite utilized in this research was supplied by Xinyang, Henan, China. The BE’s particle size distribution and XRD patterns are shown in Figure 4. Most of the particles were greater than 10 μm. The main components of the BE are shown in Table 2. The contents of SiO_2_ and Al_2_O_3_ were 69.32% and 14.27%, respectively.

(4) Fly ash (FA): The fly ash used in this research was provided by Zhengzhou, Henan, China. The specific surface area was 4300 cm^2^/g, the water demand ratio was 94.5%, and the apparent density was 2.42 g/cm^3^. The main components of the FA are shown in Table 2. The contents of SiO_2_ and Al_2_O_3_ and the loss on ignition (LOI) in FA were 55.71%, 32.79%, and 1.51%, respectively. The FA‘s particle size distribution and XRD patterns are shown in Figure 4.

### 2.2. Orthogonal Experimental Design

An orthogonal experimental design (OED) [31,32] method was used to research the performance of the DWS to obtain DWS-GM that met the actual needs. After exploring the preliminary experiments, the three materials of CE, BE, and FA were selected as the three factors. The mass ratio of the three materials was relative to the mass of the mixed DWS.

The five levels for the three factors of this experiment are shown in Table 3. L25 (53) orthogonal arrays were selected. Herein, the L is the mark of the orthogonal table. The 25 is the line number of the orthogonal table. The 5 is the level of each factor, and the 3 is the column number of the orthogonal table. A statistical analysis was carried out on the experimental results of 25 groups to acquire the influence law of various additive materials on the properties of the DWS. The orthogonal experiment scheme of this research is shown in Table S1. The initial setting time (IST), fluidity (FL), bleeding rate (BL), and mechanical properties of the drilling waste slurry grouting material (DWS-GM) were tested in this experiment.

### 2.3. Specimen Preparation and Testing Methods

**Figure 5:**
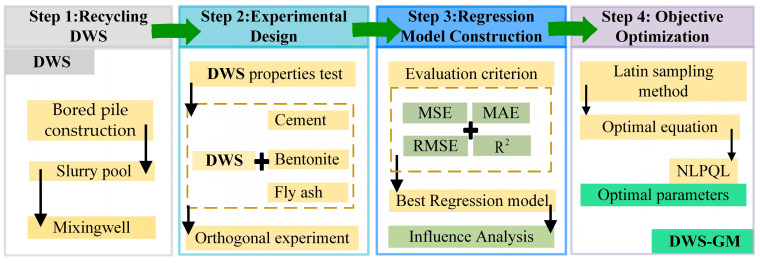
Technical route for converting reused DWS to DWS-GM.

**Figure 6:**
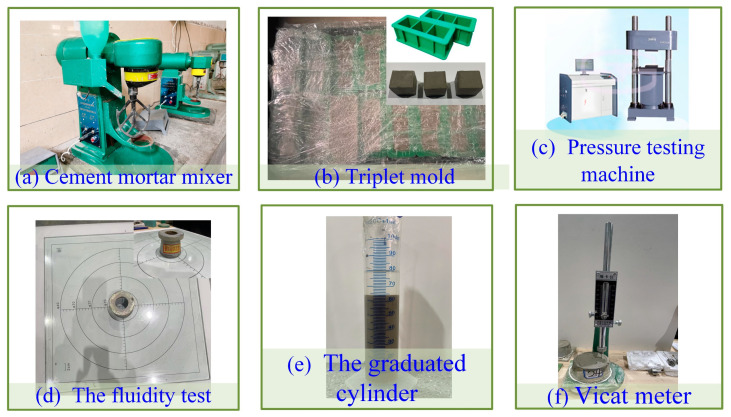
Test instruments.

The technical route of converting recycled DWS to DWS-GM is shown in Figure 5. The whole process was divided into four steps. In step 2, the preparation processes of DWS-GM were first mixed for 180 s to make the DWS evenly mixed, and three other materials (CE, BE, and FA) were subsequently mixed. To obtain the DWS-GM, a cement mortar mixer (JJ-5, Jianyi co. LTD, Wuxi, China) was used to blend the mixed powders with DWS, as shown in Figure 6a. The main experimental steps were as follows: (1) the mixing time (at a low speed) was at least 180 s; (2) stopping mixing and using a rubber scraper to scrape the paste from the mixer blades and the inside walls into the pot; and (3) the final mixing (at a high speed) lasted at least 180 s.

The DWS was then poured in triplet molds (40×40×160mm) for 3 parallel samples for each test group, as shown in Figure 6b. The molds were vibrated on a vibrating table to remove air bubbles and then covered with plastic wrap to prevent moisture loss from the DWS-GM. Specimens were then wrapped in plastic wrap and cured with relative humidity (RH 20 ± 2 °C) until they were retrieved for mechanical strength testing (7, 14, and 28 days).

The DWS-GM fluidity test [33] used the truncated conical mold (height 60 mm, top diameter 36 mm, and bottom diameter 60 mm). The size of the bottom glass plate was 400 mm × 400 mm × 5 mm. During the test, (1) the mixed DWCWS-GM was quickly poured into the truncated conical mold and (2) scraped flat with a scraper. (3) The truncated conical mold was lifted vertically, and (4) the stopwatch was started at the same time to allow the cement slurry to flow on the glass plate for 30 s. (5) The maximum diameter of the flowing part was measured in two directions perpendicular to each other with a ruler, and (6) the average value was taken as the fluidity of the DWCWS-GM.

The stability of the DWS-GM was evaluated using the bleeding rate (BL). The evenly mixed DWS-GM was put into a graduated cylinder with a fixed height. The bleed water of the DWS-GM surface was measured, as shown in Figure 6e. The ratio between the bleed water and the initial DWS-GM volume was recorded as the value of the BL [8].

According to the standard GB/T1346-2011, the initial setting time (IST) is the time from sampling to the pin penetration depth of 4 ± 1 mm from the bottom surface. The mold (height 4.5 cm, top diameter 6.5 cm, and bottom diameter 7.5 cm) is shown in Figure 6f. The test method of the DWS-GM compression strength (CS) involved placing cubes with dimensions of 40 mm × 40 mm × 40 mm in a universal testing machine (model WEW-200E, China Jinan Time Shijin Testing Machine Co., Ltd., China). The speed of the compression was controlled at 0.5 mm/min, as shown in Figure 6c. The maximum load was recorded, and the compressive strength was calculated when the specimen failed.

## 3. Analysis of Orthogonal Experiment Results 

The performance of the DWS-GM was analyzed after 25 different experimental groups were measured according to the testing method described in Section 2.3. 

### 3.1. Bleeding Rate Analysis (BL)

**Figure 7:**
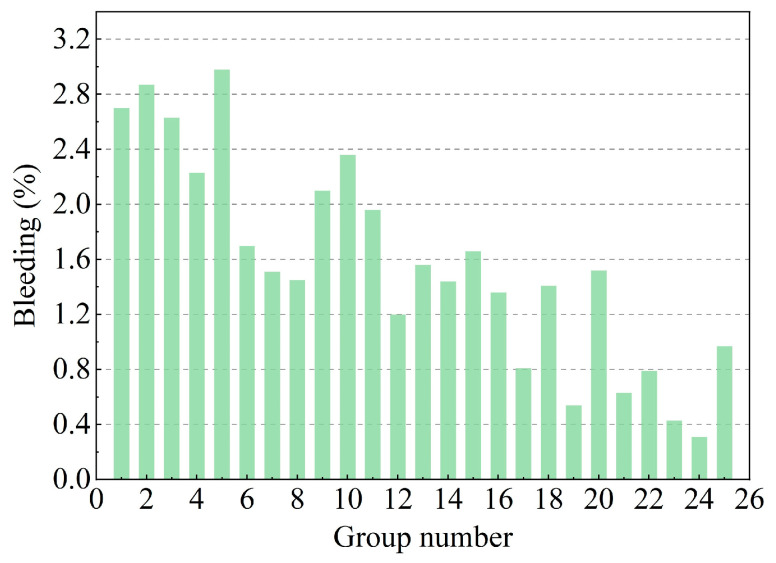
Test results of bleeding rate.

The effects of CE, BE, and FA on the bleeding rate of the DWS-GM are shown in Figure 7, The addition of CE, BE, and FA could decrease the bleeding rate. Compared with the DWS, the additives significantly improved its bleeding rate and improved the stability of the DWS-GM. CE greatly influenced the bleeding rate and could better adjust the stability of DWS-GM (groups 13, 17, and 23). Adding BE and FA reduced the bleeding rate, but the effect was insignificant (groups 1~5 and 11~15). The bleeding rate of the DWS-GM decreased from 2.98% to 0.31%. The difference between the maximum and minimum values reached about 2.67% (groups 5 and 24). The average bleeding rate was 1.56%.

**Figure 8:**
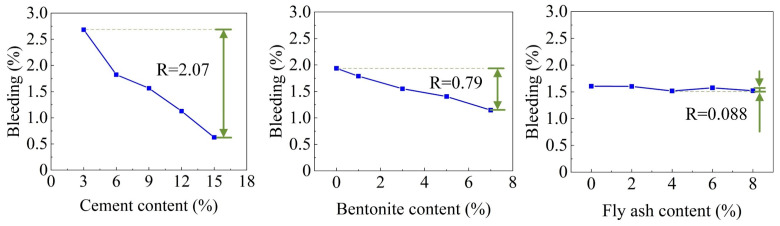
Range analysis of bleeding rate.

With an increase in the cement content, the BL of the DWS-GM further decreased. The DWS itself contained a large amount of water (the water content was 55.27%). After the CE was added, a hydration reaction occurred, and it turned into hydrates. These hydrates overlapped and were connected by various gravitational forces in a certain way to form a stable structure. In addition, BE and FA also affected the bleeding rate of the DWS. The effect on the slurry bleeding rate was much smaller than that of cement. The range analysis of each factor on the bleeding rate is shown in Figure 8. It shows that the factors (CE, BE, and FA) for the BL were ranked as follows: A > B > C. The combination of additives for the minimum BL for reusing DWS in DWS-GM was A5B5C3.

### 3.2. Fluidity Analysis (FL)

**Figure 9:**
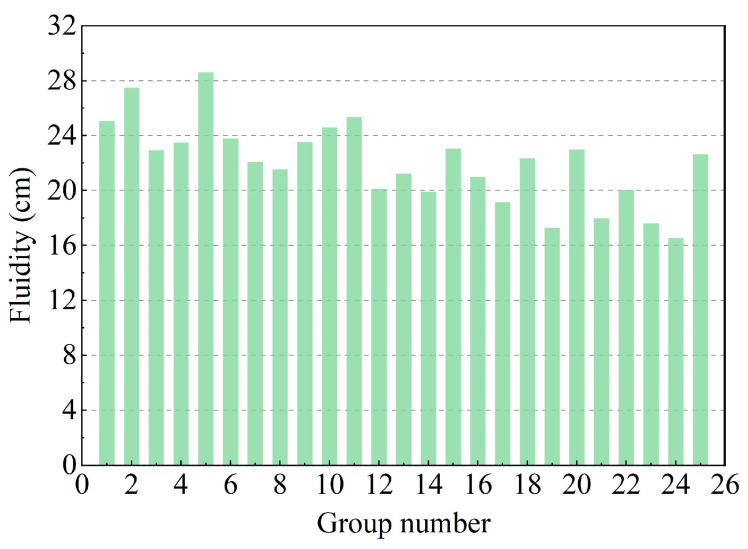
Test results of fluidity.

The fluidity requirement of the DWS-GM is as large as possible under the condition of ensuring the stability of the DWS-GM. The fluidity affects the grouting pressure and grouting efficiency to a certain extent. Excessive grouting pressure will lead to high requirements on the hardware of the entire grouting system and is not conducive to developing an automatic grouting system. A low grouting efficiency will lead to a failure to inject the grout in time, which will lead to a delay in deformation control and thus affect the overall effect of the grouting measures. As shown in Figure 9, the experimental fluidity results of the DWS-GM were generally excellent. The average fluidity was 21.9 cm. Adding a large amount of powder to the DWS caused the fluidity of the prepared DWS-GM to drop significantly (groups 3, 8, 14, 19, and 24). The fluidity of the DWS-GM decreased from 28.6 cm to 16.55 cm. The difference between the maximum and minimum values reached about 12 cm (groups 2 and 24).

**Figure 10:**
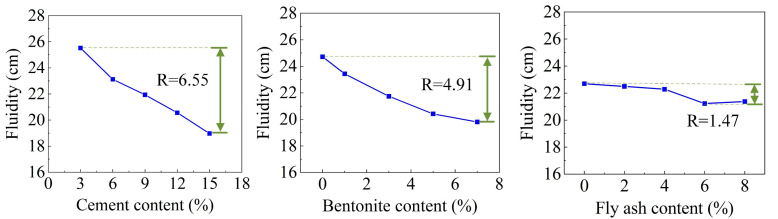
Range analysis of fluidity.

From a single-factor perspective, the increases in the CE, BE, and FA contents reduced the fluidity of the DWS, but the effect of the FA content was relatively small. The contents of CE and BE were the main factors affecting the fluidity of the DWS. The influence of CE was slightly more significant than that of BE. The range analysis of each factor on the fluidity is shown in Figure 10. It shows that the factors (CE, BE, and FA) for the FL were ranked as follows: A > B > C. The combination of additives for the maximum FL for reusing DWS in DWS-GM was A1B1C1.

### 3.3. Initial Setting Time Analysis (IST)

**Figure 11:**
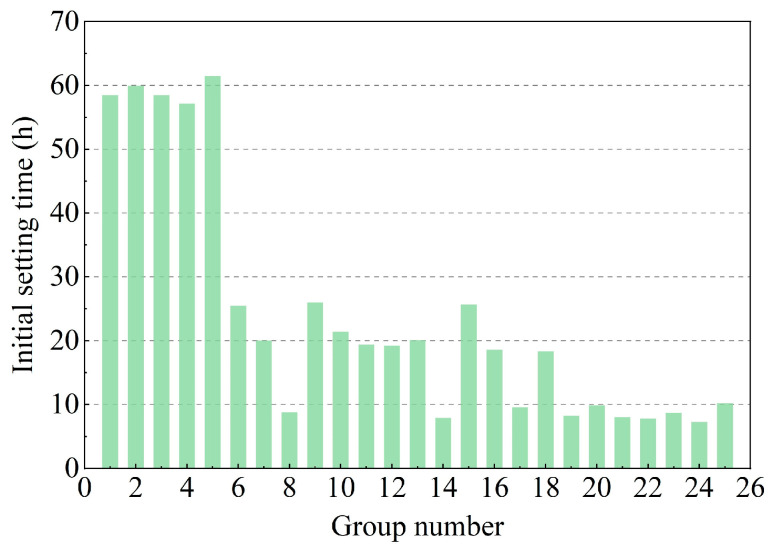
Test results of initial setting time.

It can be observed in Figure 11 that the initial setting time (IST) of the DWS-GM decreased with the cement content. The mean initial setting time with 15% cement powder content (groups 21~25) was about 8.5 h. When the cement powder content was 3% (groups 1~5), the max mean IST was about 59 h. The IST of the DWS decreased from 61.5 h to 7.28 h. The difference between the maximum and minimum values reached about 54.22 h (groups 5 and 22). The average IST was 23.97 h. The powder contents of bentonite and fly ash had little effect on the IST of the DWS-GM.

**Figure 12:**
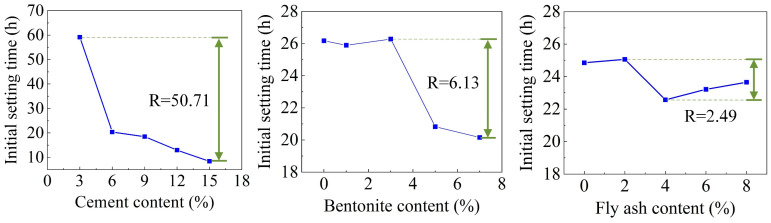
Range analysis of initial setting time.

Figure 12 illustrates the range analysis of each factor on the IST. It shows that the factors (CE, BE, and FA) for the IST were ranked as follows: A > B > C. The IST is significant for grouting, and a relatively slow IST is substantial for timely and repeated grouting control. The combination of additives for the maximum IST for reusing DWS in DWS-GM was A1B3C2.

### 3.4. Compressive Strength Analysis (CS)

**Figure 13:**
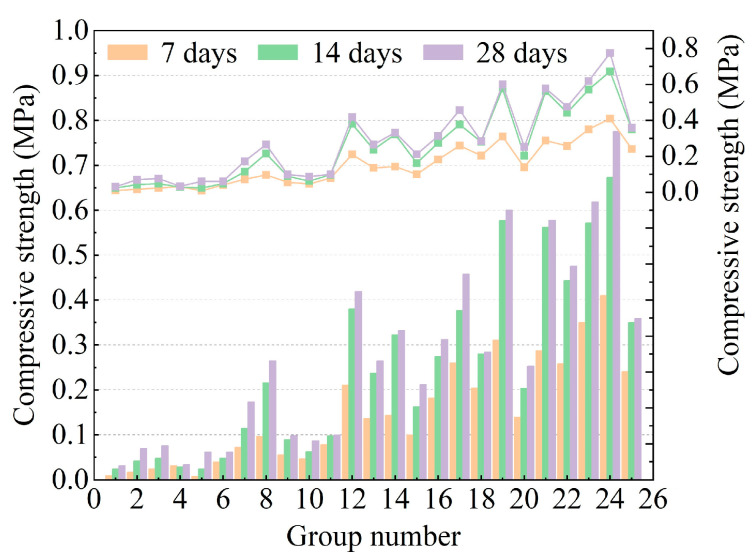
Test results of compressive strength.

Mechanical performance is related to the difficulty of multiple grouting and the grouting efficiency of the active control process. In this research, the DWS-GM specimens cured for 7 days (7DCS), 14 days (14DCS), and 28 days (28DCS) were compared to investigate their compressive strengths. The compressive strength test method is described in Section 2.3. The experimental results of different content factors on the CS of the DWS-GM are shown in Figure 13. The overall intensity of the specimens was relatively low. The max CS was 0.775 MPa when cured for 28 days. The results showed that the CS of the test pieces after curing for 7 days was significantly lower than the CS at 14 days, while the difference between 7 and 28 days was much smaller. The average CS values were 0.148 MPa, 0.248 MPa, and 0.28 MPa when cured for 7, 14, and 28 days, respectively. The mean values of the differences were 0.099 MPa and 0.032 MPa when cured for 7 and 28 days, respectively. The average CS values of the DWS were 0.054 MPa, 0.137 MPa, 0.265 MPa, 0.381 MPa, and 0.561 MPa when cured for 28 days (groups 1~25, every group of five). CE greatly influenced the CS of the DWS-GM. The difference between the maximum and minimum values reached about 0.743 MPa (groups 1 and 24).

**Figure 14:**
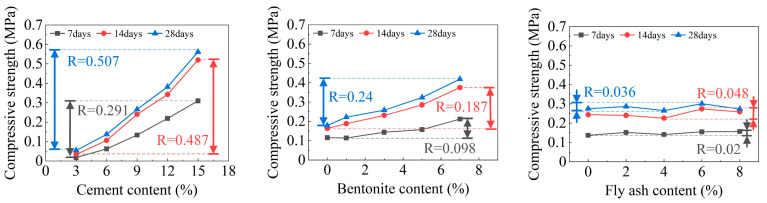
Range analysis of 7-, 14-, and 28-day compressive strengths.

With the addition of cement, the CS gradually increased. The difference between the maximum and minimum CS values varied significantly and increased with curing time. The differences in CS values when cured for 7 days, 14 days, and 28 days were 0.402 MPa, 0.648 MPa, and 0.744 MPa, respectively. The CS of the DWS-GM gradually decreased with the addition of FA. Combined with the particle size analysis results in Figure 4a, the average particle size of FA was more significant than that of BE and CE. The specific surface area was relatively large, and the activity to participate in the hydration reaction was low. A relative increase in the fly ash content led to a decrease in CS. In conjunction with Figure 7 and Figure 10, high fluidity in the DWS-GM inevitably led to low CS. The range analysis of each factor on CS is shown in Figure 14. The range of the 7-day compressive strength was the largest. The range of the 28-day compressive strength was the smallest. The compressive strength range of the DWS-GM gradually decreased with an increase in time. Compared with BE and FA, CE was the most critical factor, and its range was much higher than the others. Overall, it shows that the factors (CE, BE, and FA) for the CS were ranked as follows: A > B > C. The combination of additives for the minimum CS for reusing DWS in DWS-GM was A1B1C3.

### 3.5. Microstructure Characterization Analysis

**Figure 15:**
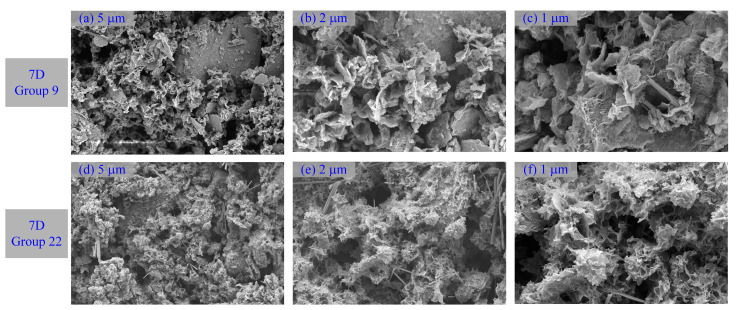
SEM photomicrographs of DWS-GM with 7 days of curing. 7D Group 9: (**a**) 2.0 KX (**b**) 5.0 KX (**c**) 10.0 KX 7D Group 22: (**d**) 2.0 KX (**e**) 5.0 KX (**f**) 10.0 KX.

**Figure 16:**
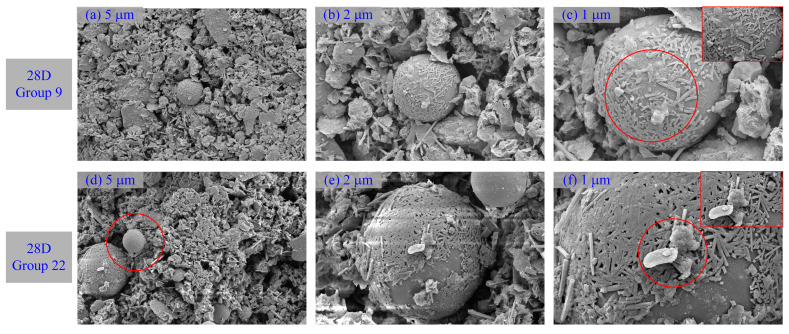
SEM photomicrographs of DWS-GM with 28 days of curing. 28D Group 9: (**a**) 2.0 KX (**b**) 5.0 KX (**c**) 10.0 KX 28D Group 22: (**d**) 2.0 KX (**e**) 5.0 KX (**f**) 10.0 KX.

Figure 15 and Figure 16 show images from SEM tests that were carried out on materials with different curing times and shooting angles to observe the morphological and pore structures inside the pattern. For the comparative analysis (Figure 3) of SEM photomicrographs of the unprocessed DWS, many fibrous and flocculent structures were generated in DWS-GM. They mainly comprised one-dimensional nanofibers, two-dimensional nanosheets, and massive particles of various shapes and sizes. Two-dimensional nanosheets were coated on the surfaces of larger bulk particles, and nanofibers were distributed between the nanosheets. Micro- and nanostructures of different sizes and dimensions were assembled, showing a multilevel composite morphology structure.

One-dimensional nanofibers had diameters of several hundred nanometers and lengths of several micrometers, while nanosheets had lateral dimensions of several hundred nanometers. In addition, due to the accumulation caused by the interactions between the two-dimensional nanosheets, there were more apparent wrinkles, which provided a rich pore structure. These structures were irregularly distributed in the intergranular pores, enveloped the mud particles, and then aggregated together to form a sheet structure. This implied that a relatively robust structure had been created.

A sphere was considered an FA particle, as shown in Figure 16. Many delicate structures were bonded and wrapped on the surfaces of FA particles. It can be observed that many nanorods were tightly coated on the surfaces of spherical particles. The diameters of these nanorods were about 100 nanometers, and the lengths were about 1 micron. This may have been due to the existence of chemical bonds or strong bonds between nanorods and spherical particles. The interactions led to the assembly of nanorods and spherical particles to form new composite structures.

## 4. Experimental Regression Analysis

### 4.1. Regression Models

This section describes regression models based on the experimental results of the above orthogonal experiments (groups 1~25). The effects of CE, BE, and FA on the performance of DWS-GM were constructed by the regression functions (RM). After a large amount of data analysis and model comparison, three different base models, including a linear model (LM), a support vector machine (SVM), and a neural network (NN), were selected to analyze the performance of the DWS-GM. The root-mean-square error (RMSE), mean absolute error (MAE), coefficient of determination (R2), and mean square error (MSE) were used as the evaluation criteria for the model [34,35].

**Figure 17:**
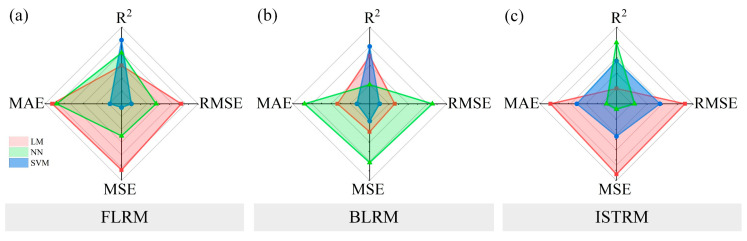
Performances of FL, BL, and IST regression models. (**a**) FLRM (**b**) BLRM (**c**) ISTRM.

**Figure 18:**
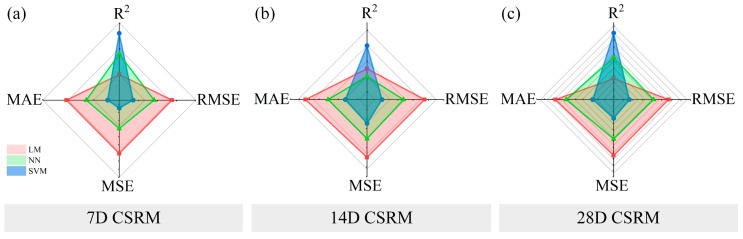
Performances of CS regression models. (**a**) 7D CSRM (**b**) 14D CSRM (**c**) 28D CSRM.

The performances of the regression models are shown in Table S2. Compared with other models generated in the process, the coefficients of determination of the above models were all larger than 0.9. The other three evaluation criteria were minimal. In Figure 17 and Figure 18, a comparative analysis of the three models is described. The figures present the performances of the regression models on radar charts to visualize the performances more intuitively.

From the perspective of the four evaluation indicators, the larger the R2, the smaller the RMSE, MAE, and MSE, and the model had the best performance. It can be seen from the figure that the regression model with a smaller occupied area had the best performance. The fluidity (FLRM), bleeding rate (BLRM), and 28-day compressive strength regression models (28D CSRM) were all SVMs, and the kernel function was a cubic polynomial. The 7- and 14-day compressive strength regression models (7D CSRM and 14D CSRM) were both SVMs, but their kernel functions were quadratic polynomials. The initial setting time regression model (ISTRM) was a NN, and the layer number was three.

**Figure 19:**
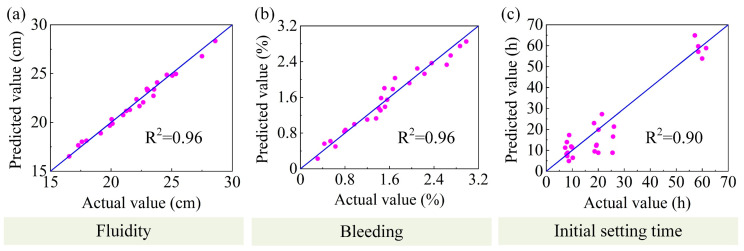
Performances of FL, BL, and IST predicted models. (**a**) FL (**b**) BL (**c**) IST.

**Figure 20:**
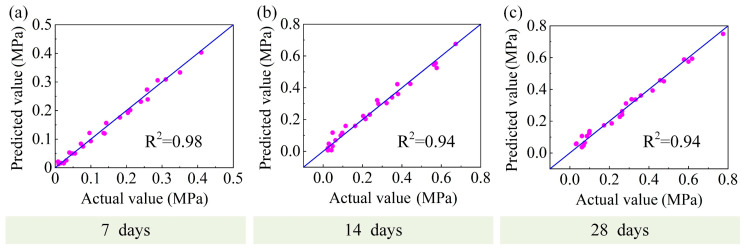
Performances of CS predicted models. (**a**) 7D (**b**) 14D (**c**) 28D.

Scatter plots between the actual and predicted values for the models during the training phase are presented in Figure 19 and Figure 20. The predicted and actual values were in good agreement. It was fully demonstrated that the above model was sufficient to describe the performance of the DWS-GM within a specific range. The specific parameters of the final optimal performance regression model are shown in Table S3.

### 4.2. Regression Analysis

**Figure 21:**
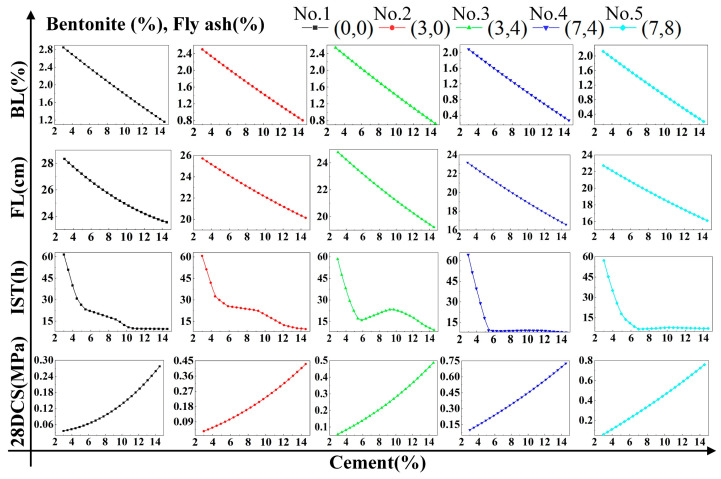
Influence of CE content on DWS-GM.

To research the effect the single factor on DWS-GM, the influence of the cement is shown in Figure 21. When only cement was added to change the DWS, the bleeding rate, fluidity, and initial setting time showed rapid downward trends. Among them, the initial setting time stabilized when the content reached 10%. The 28-day compressive strength of the DWS-GM increased with the addition of cement content. After adding bentonite and fly ash, the overall change trends of various performance indicators of the DWS did not change. After adding bentonite, the bleeding rate and fluidity had relatively apparent decreases (groups 1 and 2 and groups 3 and 4 in Figure 21). The increase in fly ash had little effect on various properties (Groups 2 and 3 and groups 4 and 5 in Figure 21). For the initial setting time, adding a certain amount of bentonite reduced the impact of cement on the IST. The addition of fly ash had a specific promoting effect on the IST and CS.

**Figure 22:**
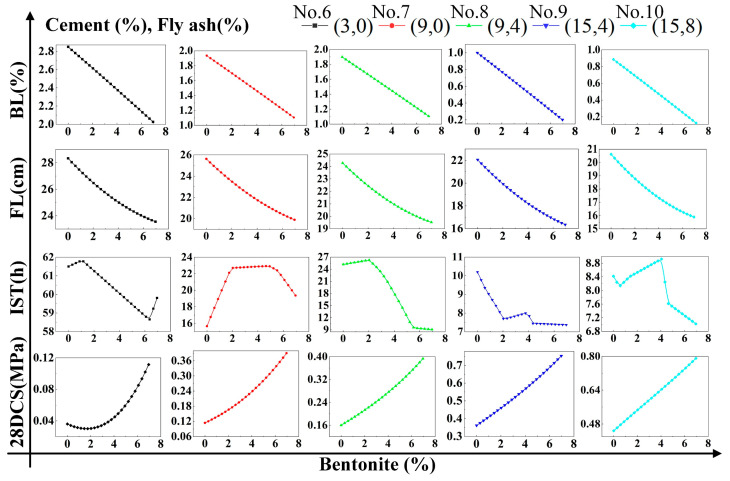
Influence of BE content on DWS-GM.

**Figure 23:**
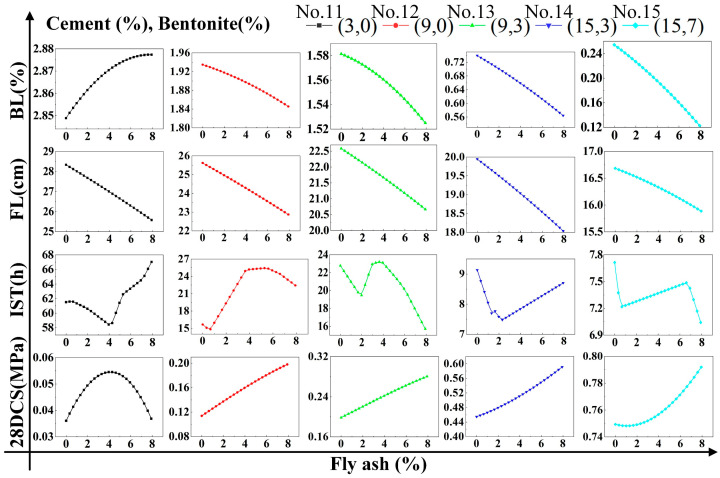
Influence of FA content on DWS-GM.

Combined with the observation in Figure 22, the bleeding rate and fluidity decreased gradually with the increase in bentonite. The addition of cement and fly ash slowed the rate at which bentonite caused decreases in the fluidity and bleeding rate. The initial setting time showed a large fluctuation. It can be seen in Figure 23 that a small amount of fly ash could increase the bleeding rate of the DWS-GM to a certain extent, and as analyzed in Section 3.1, fly ash prevented the hydration reaction to a certain extent. However, with the addition of the other two substances, this effect gradually weakened. The addition of fly ash resulted in slight decreases in the fluidity and bleeding rate (groups 11 and 13 in Figure 23). The influence of bentonite and fly ash on the IST fluctuated wildly, and the trend was not apparent, but the overall impact was relatively small. In the comparative analysis (Figure 21, Figure 22 and Figure 23), the 28DCS of the DWS-GM increased with increases in bentonite and fly ash. However, compared with the influence of the cement content, it was not a critical influencing factor.

## 5. Optimization

After the above analysis, the influence of each factor on the DWS was explored in detail. To further investigate better formula proportions to obtain better DWS-GM, more experimental groups were extracted for multi-objective optimization in the design space of the above orthogonal experiments, as shown in Table 4. During sampling, the hyper-Latin sampling method (HLS) [36,37] was adopted to ensure the uniformity of the sample extraction.

**Figure 24:**
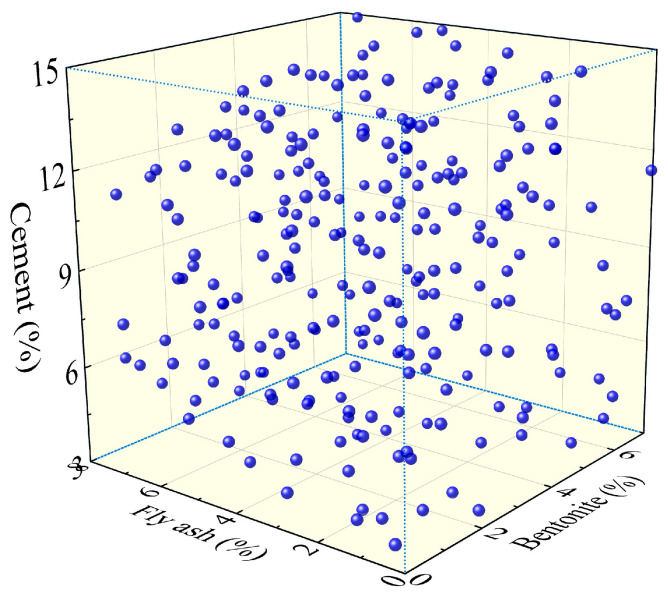
Experimental samples for the hyper-Latin sampling method (HLS).

As shown in Figure 24, 250 groups of experimental samples were extracted to form the experimental data set for objective optimization. The design space is depicted within the ranges of the three coordinate axes. The dots in it represent the extracted samples. Herein, no specific experiments were carried out. The values of various performance indicators of the DWS-GM were calculated using the regression performance model in Section 4.1.

The fitted optimal equation describes the performance of the DWS-GM, which is related to the variables by the third-order polynomial (Equation (1)) below:

(1) $$f_{p}(x)=a_{0}+\sum_{i=1}^{t}b_{i}x_{i}+\sum_{i=1}^{t}c_{i}x_{i}^{2}+\sum_{i=1}^{t}\sum_{i<j}^{t}d_{i,j}x_{i}x_{j}+\sum_{i=1}^{t}e_{i}x_{i}^{3}$$

where $f_{p}(x)$ is the performance of the DWS-GM; $x_{i}$ and $x_{j}$ are the independent variables; $a_{0}$, $b_{i}$, $c_{i}$, and $e_{i}$ are the different coefficients; $x_{i}x_{j}$ represents the cross term of the parameter coupling relationship; *x_CE_* is the value of the cement content; $x_{BE}$ is the value of the bentonite content; and $x_{FA}$ is the value of the fly ash content.

The fitted third-order polynomial results are shown in Table S4. According to the preceding analysis and discussion, the optimal target values were determined as the bleeding rate, fluidity, initial setting time, and 28-day compressive strength. The 28-day intensity was more representative. The following four aspects of the optimal combination of additives were considered: (1) minimizing the bleeding rate; (2) maximizing the fluidity of the DWS-GM; (3) maximizing the initial setting time; and (4) maximizing the 28-day compressive strength. The objective function is given as follows:

(2) $$f_{OPT}(x)=\left\{\min[f_{BL}(x)],\max[f_{FL}(x)],\min[f_{IST}(x)],\max[f_{28}{}_{CS}(x)]\right\}$$

where $f_{OPT}(x)$ is the optimal equation of the DWS-GM and $f_{BL}(x)$, $f_{F}{}_{L}(x)$, $f_{IST}(x)$, and $f_{28}{}_{CS}(x)$ are the performance values of the bleeding rate, fluidity, initial setting time, and 28-day compressive strength.

Thus, other constraints needed to be added to ensure the recipe was realistic. Combined with the actual analysis, all performance indicators needed to be greater than 0. The 28DCS was more significant than 0.1 MPa, according to the experience during the experiment. The fluidity needed to be greater than 14.8 cm [7]. The bleeding rate was less than 1.61% [8]. The objective constraints are given as follows:

(3) $$\{\begin{array}{c} 0<f_{BL}(x)<1.61\\ 14.8<f_{FL}(x)\\ 0<f_{IST}(x)\\ 0.1<f_{28}{}_{CS}(x) \end{array}$$

The non-linear programming by the quadratic Lagrangian program (NLPQLP) [38] method was used to obtain a unique solution by solving the above objective optimizations. NLPQLP is a special implementation of the sequential quadratic programming (SQP) method. The ISIGHT optimization toolbox was used to obtain the optimal formulation. The optimal parameters of DWS in this paper were a cement content of 0.056 and a bentonite content of 0.069. The optimization results showed that fly ash can be considered unnecessary. The performance parameters of the obtained DWS-GM, in this case, were a bleeding rate of 1.61%, a fluidity of 21.87 cm, an initial setting time of 27.05 h, and a 28-day compressive strength of 0.22 Mpa.

## 6. Conclusions

The primary research purpose of this paper was to reuse the waste slurry dis-charged from bored piles and to meet the requirements for engineering application and green environmental protection. The properties of the drilling waste slurry (DWS) were modified by adding cement (CE), bentonite (BE), and fly ash (FA), and the DWS was reused for the active control of real-time capsule grouting technique (RCG). The main conclusions can be summarized as follows:

(1) DWS is a material with high fluidity. Its high bleeding rate indicates poor stability. The microscopic two-dimensional flake structure provides a higher specific surface area.
(2) CE had a significant influence on the properties of the DWS, while BE and FA played important auxiliary roles. High CE, BE, and FA contents could increase the CS and decrease the BI, FL, and IST of the DWS-GM.
(3) The optimal formulation of the DWS-GM treatment recommends a CE content of 0.056, a BE content of 0.069, and no added fly ash under the objective constraints of the design.
(4) The optimal material ratio and performance of the DWS-GM applied to RCG was obtained with a fluidity of 21.87 cm, a bleeding rate of 1.61%, an initial setting time of 27.05 h, and a 28-day compressive strength of 0.22 MPa.
(5) The reused DWS not only reduces project costs but also promotes sustainable and clean raw materials. The application of multifunctional materials, such as high-strength and retarded materials based on DWS, will be further developed.

## Figures and Tables

**Table 1:** The main properties of the DWS.

Item	Values
Wet density (g/cm^3^)	1.45
Water content (%)	55.27
Fluidity (cm)	31.2
Bleeding rate (%)	26.74

**Table 2:** The compositions of CE, BE, and FA.

Chemical Composition		SiO_2_	Al_2_O_3_	CaO	MgO	Fe_2_O_3_	Na_2_O	K_2_O	P_2_O_5_	SO_3_	Others	LOI
Content	Cement (CE)	24.99	8.26	51.42	3.71	4.03	/	/	/	2.51	1.77	3.31
(wt%)	Bentonite (BE)	69.32	14.27	1.99	2.69	1.84	1.58	1.04	0.04	/	0.56	5.67
	Fly ash (FA)	55.71	32.79	2.656	0.235	4.429	/	1.541	/	0.65	0.479	1.51

**Table 3:** The orthogonal experiment (Wt%).

Factor Levels	Cement (CE) (%)	Bentonite (BE) (%)	Fly Ash (FA) (%)
1	3	0	0
2	6	1	2
3	9	3	4
4	12	5	6
5	15	7	8

**Table 4:** Decision variables.

Factors	Variable	Type	Value Range
Cement	$x_{CE}$	Continuous	[0.03,0.15]
Bentonite	$x_{BE}$	Continuous	[0,0.07]
Fly ash	$x_{FA}$	Continuous	[0,0.08]
Cement	$x_{CE}$	Continuous	[0.03,0.15]
Bentonite	$x_{BE}$	Continuous	[0,0.07]

## Supplementary Materials

The following supporting information can be downloaded at https://www.mdpi.com/article/10.3390/ma16041540/s1, Table S1: Orthogonal experiment proportions (Wt%); Table S2: Parameters of the regression models; Table S3: Performances of regression models; Table S4: Results of fitted optimal equation.
