# Supplementary material for: Reuse of Drilling Waste Slurry as the Grouting Material for the Real-Time Capsule Grouting Technique
Source: Materials (Basel). 2023 Feb 12;16(4):1540. doi: 10.3390/ma16041540 (PMC9962331; doi:10.3390/ma16041540)
Supplement: Supplementary file 1 [file materials-16-01540-s001.zip › materials-2186711-supplementary.pdf]

Table S1. Orthogonal experiment proportions (Wt%).

Experiment number	Abbreviation	Cement (%)	Bentonite (%)	Fly ash (%)
		A	B	C
1	A1B3C3	3	3	4
2	A1B2C2	3	1	2
3	A1B4C4	3	5	6
4	A1B5C5	3	7	8
5	A1B1C1	3	0	0
6	A2B3C1	6	3	0
7	A2B 4C2	6	5	2
8	A2B5C3	6	7	4
9	A2B2C5	6	1	8
10	A2B1C4	6	0	6
11	A3B1C2	9	0	2
12	A3B5C1	9	7	0
13	A3B3C4	9	3	6
14	A3B4C5	9	5	8
15	A3B2C3	9	1	4
16	A4B2C1	12	3	2
17	A4B4C4	12	5	4
18	A4B1C5	12	0	8
19	A4B5C4	12	7	6
20	A4B2C1	12	1	0
21	A5B4C1	15	5	0
22	A5B2C4	15	1	6
23	A5B3C5	15	3	8
24	A5B5C2	15	7	2
25	A5B1C3	15	0	4

Table S2. Performance of regression models.

	Model	RMSE	R^2	MSE	MAE
FL	LM	0.9122	0.92	0.8321	0.6809
	**SVM**	**0.6515**	**0.96**	**0.4245**	**0.5303**
	NN	0.7810	0.94	0.61	0.6697
BE	LM	0.1861	0.95	0.0347	0.1467
	**SVM**	**0.1703**	**0.96**	**0.0290**	**0.1366**
	NN	0.2252	0.92	0.0507	0.1639
IST	LM	12.959	0.60	167.94	10.893
	SVM	9.6912	0.78	93.919	8.1511
	**NN**	**6.3628**	**0.90**	**40.485**	**5.0402**
7 days CS	LM	0.0376	0.90	0.0014	0.0307
	**SVM**	**0.0173**	**0.98**	**0.0003**	**0.0146**
	NN	0.0283	0.94	0.0008	0.0229
14 days CS	LM	0.0775	0.91	0.0060	0.0620
	**SVM**	**0.0498**	**0.94**	**0.0025**	**0.0413**
	NN	0.0638	0.90	0.0041	0.0503
28 days CS	LM	0.0971	0.81	0.0094	0.0679
	**SVM**	**0.0564**	**0.94**	**0.0032**	**0.0436**
	NN	0.0816	0.87	0.0067	0.061

Table S3. Parameters of the regression models.

$f_p(x)$	Models	Models Detailed description
$f_{BL}(x)$	FLRM	Cubic kernel function, Box Constraint: 997.206, Epsilon: 0.132
$f_{FI}(x)$	BLRM	Quadratic kernel function, Box Constraint: 2.669, Epsilon: 0.267
$f_{IST}(x)$	ISTRM	Three-layer Neural Network, LayerSizes:10, Activation function: ReLU
	7D CSRM	Quadratic kernel function, Box Constraint: 0.024, Epsilon: 0.0085
$f_{CS}(x)$	14D CSRM	Quadratic kernel function, Box Constraint: 0.169, Epsilon: 0.0182
	28D CSRM	Cubic kernel function, Box Constraint: 0.255, Epsilon: 0.0255

Table S4. Results of fitted optimal equation.

$f_P(x)$	Third-order polynomial fitted equation
$f_{BL}(x)$	$f_{BL}(x) = 3.333 - 16.465x_{CE} - 11.524x_{BE} + 1.481x_{FA} + 10.22x_{CE}^2 - 3.539x_{BE}^2 - 4.69x_{FA}^2 + 0.558x_{CE}^3 - 1.262x_{BE}^3 + 0.042x_{FA}^3 - 1.616x_{CE}x_{BE} - 24.861x_{CE}x_{FA} + 13.78x_{BE}x_{FA}$
$f_{FL}(x)$	$f_{FL}(x) = 29.878 - 53.443x_{CE} - 94.877x_{BE} - 32.421x_{FA} + 67.882x_{CE}^2 + 474.602x_{BE}^2 - 30.602x_{FA}^2 + 0.028x_{CE}^3 - 0.05x_{BE}^3 - 0.113x_{FA}^3 - 227.562x_{CE}x_{BE} + 1.732x_{CE}x_{FA} + 350.353x_{BE}x_{FA}$
$f_{IST}(x)$	$f_{IST}(x) = 134.024 - 3559.398x_{CE} + 334.006x_{BE} - 17.034x_{FA} + 35143.959x_{CE}^2 - 12103.34x_{BE}^2 - 725.495x_{FA}^2 - 115737.197x_{CE}^3 + 110742.354x_{BE}^3 + 7027.7x_{FA}^3 + 193.279x_{CE}x_{BE} + 1371.423x_{CE}x_{FA} - 3484.376x_{BE}x_{FA}$
$f_{28CS}(x)$	$f_{28CS}(x) = 0.0197 - 0.247x_{CE} - 0.714x_{BE} + 0.134x_{FA} + 15.281x_{CE}^2 + 3.749x_{BE}^2 + 2.809x_{FA}^2 - 16.258x_{CE}^3 + 113.729x_{BE}^3 - 33.609x_{FA}^3 + 41.837x_{CE}x_{BE} + 12.535x_{CE}x_{FA} - 14.294x_{BE}x_{FA}$
